# Long‐term obesogenic diet and targeted deletion of potassium channel K_v_1.3 have differing effects on voluntary exercise in mice

**DOI:** 10.14814/phy2.14254

**Published:** 2019-10-23

**Authors:** Brandon M. Chelette, Abigail M. Thomas, Debra Ann Fadool

**Affiliations:** ^1^ Department of Biological Science The Florida State University Tallahassee Florida; ^2^ Programs in Neuroscience The Florida State University Tallahassee Florida; ^3^ Molecular Biophysics The Florida State University Tallahassee Florida

**Keywords:** Obesity, running, wheel

## Abstract

Voluntary exercise is frequently employed as an intervention for obesity. The voltage‐gated potassium channel K_v_1.3 is also receiving attention as a therapeutic target for obesity, in addition to potential therapeutic capabilities for neuroinflammatory diseases. To investigate the combinatorial effects of these two therapies, we have compared the metabolic status and voluntary exercise behavior of both wild‐type mice and a transgenic line of mice that are genetic knockouts for K_v_1.3 when provided with a running wheel and maintained on diets of differing fat content and caloric density. We tracked the metabolic parameters and wheel running behavior while maintaining the mice on their assigned treatment for 6 months. Wild‐type mice maintained on the fatty diet gain a significant amount of bodyweight and adipose tissue and display significantly impaired glucose tolerance, though all these effects were partially reduced with provision of a running wheel. Similar to previous studies, the K_v_1.3‐null mice were resistant to obesity, increased adiposity, and impaired glucose tolerance. Both wild‐type and K_v_1.3‐null mice maintained on the fatty diet displayed increased wheel running activity compared to control‐fed mice, which was caused primarily by a significant increase in the amount of time spent running as opposed to an increase in running velocity. Interestingly, the patterns of running behavior differed between wild‐type and K_v_1.3‐null mice. K_v_1.3‐null mice spent significantly less time running during the light phase and displayed a decrease in running 1–2 h before the onset of the light phase, seemingly in anticipation of the dark‐to‐light phase transition. These studies indicate that voluntary exercise combats metabolic maladies and running behavior is modified by both consumption of an obesogenic diet and deletion of the K_v_1.3 channel.

## Introduction

The prevalence of obesity in the human population is increasing at an alarming rate. In the United States, the percentage of obese adults has increased from 15% in 1990 to over 35% in 2012 and obesity is now commonly described as an epidemic (Ogden et al. 2014). Obesity has been correlated with increased incidence of many diseases as well as increased mortality (Smith et al. 2016). Financial impact estimates vary, but the cost of obesity is reported in the hundreds of billions of dollars in the United States (Smith et al. 2016). These troubling trends have necessitated an increased research effort to uncover prevention and treatment strategies for obesity.

Exercise has long been prescribed to treat obesity, but the benefits of engaging in voluntary exercise extend beyond attempts to lose weight (Dipietro 1999). Studies have indicated that participation in voluntary exercise suppresses inflammation in the central nervous system, slows the cognitive decline and reduction in hippocampal volume associated with aging, modulates the microbiome, and combats insulin resistance (Yuede et al. 2009; Do et al. 2018; Allen et al. 2018). Conversely, inactivity has been identified as a risk factor for the development of neurodegenerative diseases such as Alzheimer’s disease and dementia (Norton et al. 2014). In light of the obesity epidemic, the more recent discovery of the additional health benefits of exercise, and the detriments of a sedentary lifestyle, voluntary exercise is receiving renewed research attention (Patten et al. 2015; Idorn et al. 2017).

Another promising therapeutic target is the voltage‐gated potassium channel, K_v_1.3. Potassium ion channels are one of the largest families of ion channels. Potassium channels are historically characterized to function as dampeners of excitability through shaping of the action potential and timing of the interspike interval (Hille 2001). We now know there are multifarious roles of potassium channels that include a number of nonconductive functions such as their ability to regulate or detect energy substrates, to be key players in immune responses, and to modulate sensory perception (Fadool et al. 2004; Gocke et al. 2012; Tucker et al. 2013). K_v_1.3 is a mammalian homolog of the *Shaker* subfamily that has a select distribution within the CNS (the olfactory bulb, the pyriform cortex, and the dentate gyrus of the hippocampus), the peripheral organs (adipocytes, kidney), and the immune system (T‐cells) (Cahalan et al. 1985; Kues and Wunder 1992; Fadool et al. 2000; Fadool et al. 2004; Wang 2004; Li et al. 2006; Al Koborssy et al. 2019). Efforts by our laboratory and others have revealed that K_v_1.3‐null mice exhibit several interesting metabolic phenotypes. Multiple studies have shown that K_v_1.3‐null mice are thin compared with wild‐type mice, have a resistance to diet‐induced obesity (DIO), and exhibit a slightly increased basal metabolic rate (Xu et al. 2003; Xu et al. 2004; Tucker et al. 2008). Furthermore, the blockade of K_v_1.3 increases glucose uptake by increasing the trafficking of glucose transporter type 4 (GLUT4) to the membrane of skeletal muscle cells and adipocytes (Li et al. 2006). Gene deletion or pharmacological blockade of K_v_1.3 has been shown to reduce neuroinflammation and has been used to treat a range of animal model versions of autoimmune diseases including multiple sclerosis, psoriasis, and type‐1 diabetes (Chandy and Norton 2017). The treatments have been effective and researchers have been successful in increasing the potency, selectivity, and targeting of K_v_1.3 blockers (peptides isolated from scorpion and sea anemone toxins) (Rashid et al. 2013). Due to this unique combination of physiological effects driven by K_v_1.3, the channel has become an important and promising therapeutic target for neuroinflammatory diseases, metabolic disorders, and neurodegeneration (Pérez‐Verdaguer et al. 2016; Serrano‐Albarras et al. 2018).

The most promising therapeutic directions for both voluntary exercise and K_v_1.3‐targeted treatments are converging on a subset of human diseases for which exercise or changed metabolism might be effective. This is especially true for diseases that have both a metabolic and autoimmune component. Progress is being made rapidly in both areas of research, but little is known concerning the potential combinatorial effects of these two therapeutic interventions. This is an important consideration since other treatments targeted toward metabolic syndrome have shown negative interactions with exercise behavior. Metformin and rosiglitazone, two commonly prescribed anti‐diabetic medications, have been shown to reduce exercise capacity (Paul et al. 2017; Bastien et al. 2019). Some patients taking SGLT2 inhibitors to help lower their blood sugar have experienced diabetic ketoacidosis during bouts of increased physical activity (Peters et al. 2015). Although the biophysical properties of K_v_1.3 in many cells are well characterized and the channel has become an important therapeutic target as outlined above, less is known concerning how loss of K_v_1.3 may affect activity patterns or if voluntary running is enhanced in the absence of the channel. The changes in locomotor activity reported in previous studies focus on spontaneous physical activity (ambulatory movement in home cage, grooming, etc.) and obligatory movement (food/water seeking, food/water consumption, etc.). Wheel running is a voluntary movement and these different categories of activity contribute differently to total energy expenditure and are influenced differently by genetic and environmental factors (Garland et al. 2011).

Our investigation is the first to characterize the running phenotype of mice where we measured the interactions of six‐month access to voluntary exercise, diet modification, and genetic loss of the K_v_1.3 channel. We found that both wild‐type and K_v_1.3‐null mice challenged with a moderately high‐fat diet participated in wheel running more often and ran farther than control‐fed mice. We also demonstrated that participation in voluntary exercise, as expected, partially protected wild‐type mice from increased adiposity and glucose insensitivity associated with the MHF diet when compared with MHF‐fed sedentary mice. Finally, K_v_1.3‐null mice ran the same distance and velocity as wild‐type mice when maintained on the same diet, but their patterns of resting and running differed across the both the light and dark phases.

## Materials and Methods

### Animal care

All mice were maintained in the Florida State University (FSU) animal vivarium in accordance with the institutional requirements established by the FSU Animal Care and Use Committee (ACUC) and all experimental procedures were performed according to the approved FSU ACUC protocol #1733. Our laboratory and others have previously demonstrated that female mice either did not gain adiposity or gained it at a much slower rate than male mice, therefore only male mice were used in this current investigation to explore the intersection of diet, voluntary exercise, and presence/absence of K_v_1.3 channel (Yang et al. 2014; Chelette et al. 2018). Mice were weaned to large, breeding‐style cages (18 × 9.25 × 6.25 cm) at postnatal day (PND) 21‐25 to accommodate the running wheel equipment (see **Voluntary Exercise Behavior** section for a description of the wheel equipment). The mice were maintained on either a normal rodent chow diet (control fed, CF; LabDiet 5001 Rodent Chow; 13.5% kcal from fat, https://www.labdiet.com/Products/StandardDiets/Rodents/index.html) or a moderately high‐fat, condensed milk diet (MHF; Research Diets, Cat #D12266B, 31.8% kcal from fat, https://researchdiets.com/formulas/d12266b). The selection of the dietary treatment was randomly assigned. The mice were singly housed in conventional cages upon weaning, provided ad libitum access to food and water, maintained on a 12 h/12 h light/dark cycle, and provided only their assigned diet for the duration of the experimental period (~160 days). All mice were provided two additional sources of enrichment that included a house and nesting squares.

### Animal lines

To investigate the role of the ion channel K_v_1.3 on exercise behavior, a transgenic line of mice that were global genetic knockouts for K_v_1.3 (K_v_1.3‐null) were used. These mice were on a C57BL/6J background and were generated via deletion of a large promoter region as well as the N‐terminal third of the coding sequence for K_v_1.3 (Koni et al. 2003; Xu et al. 2003). The mice were a generous gift of Drs. Leonard Kaczmarek and Richard Flavell (Yale University, New Haven, CT) and have now been deposited at Jackson Laboratories (Bar Harbor, ME; B6;129S1‐Kcna3tm1Lys/J, stock number 027392, https://www.jax.org/strain/027392, RRID: MGI:2679442). The K_v_1.3‐null mice (KO) were compared to mice with a wild‐type (WT) version of the channel. The wild‐type cohort consisted of two lines of mice. The first were C57BL/6J mice from Jackson Laboratories (stock number 000664, https://www.jax.org/strain/000664, RRID: IMSR_JAX:000664). The second were *M72‐IRES‐tau‐Lac* mice (gene *oflr160*) on a C57BL/6J background. The only difference between the C57BL/6J and the *M72‐IRES‐tau‐LacZ* mice was an addition of a genetic reporter in the latter, which allows for the visualization of a subset of olfactory sensory neurons (Mombaerts et al. 1996; Zheng et al. 2000; Biju et al. 2008). The *M72‐IRES‐tau‐LacZ* mice were generously provided by Dr. Peter Mombaerts (Max‐Planck‐Gesellschaft, Munich, Germany, now deposited at Jackson Laboratories, stock number 006596, https://www.jax.org/strain/006596, RRID: IMSR_JAX:006596) and were used here with the intention of applications for future experiments to investigate the anatomy of the olfactory system following voluntary exercise and pair feeding (not performed here) (Chelette et al. 2018). Several investigators have shown that voluntary exercise is highly variable and certain transgenic lines run abnormal amounts when given access to a wheel (Castilla‐Ortega et al. 2013). However, there were no differences between the running behavior (or any other metrics) of the C57BL/6J versus the *M72‐IRES‐tau‐LacZ* mice, so these two groups were pooled and referred to as wildtype (WT) throughout this manuscript.

### Bodyweight and adiposity

To compare normal weight gain to the increased weight gain and deposition of adipose tissue caused by maintenance on the MHF diet, the bodyweight of each mouse was recorded weekly. Upon termination, fat pads were excised and weighed by an investigator that was blind to the genotype and treatment group of the mouse. Epididymal, retroperitoneal, subcutaneous (a subsample of the right side), and mesenteric adipose tissues were collected and combined. Brown fat was not sampled.

### Voluntary exercise behavior

Mice belonging to the voluntary exercise treatment groups were provided ad libitum access to a running wheel in their cage. Upon weaning and following a 2‐day acclimation period to their larger, individual cage, a Vertical Wireless Running Wheel (Med Associates, Inc., St Albans, VT, https://www.med-associates.com/product/vertical-wireless-running-wheel-for-mouse/) was affixed in the cage. The wheel sensor was placed atop the wire lid and plastic manifolds extended into the cage that held the running wheel above the bedding and allowed for rotation. A clean wheel was provided weekly and the wheels were lubricated with non‐caloric, tasteless, and odorless silicone oil as necessary to minimize potentially stressful squeaking of the rotating wheel. The wheel sensor recorded the number of rotations and transmitted the data to a central hub every 30 sec (s). The hub was in turn connected to a computer equipped with the Wheel Manager software program (Med Associates, Inc., https://www.med-associates.com/product/wheel-manager/), which archived the data and allowed for data exportation upon conclusion of the running period. The running data were exported to Microsoft Excel (Microsoft Office 365, https://www.microsoft.com/en-us/education/products/office) via the Wheel Analysis software program (Med Associates, Inc.). A 1‐minute bin was the smallest bin size that the software allowed to be exported.

A majority of the running behavior data reported in this manuscript are a 28‐day subsample for each mouse instead of the entire experimental period of 6 months. We have concluded that restricting analysis to a 28‐day period is justifiable because (1) it allowed for the exclusion of the 3‐day acclimation period during which mice ran very little and/or inconsistently and (2) it allowed for the exclusion of outliers in running behavior that were obviously due to a technical error (trapping of bedding or low battery) as opposed to actual behavioral changes of a mouse. Due to staggered breeding, not all mice initiated the exercise treatment on the same calendar date and were therefore differentially affected by software malfunctions, dead batteries, or extreme weather conditions requiring evacuation by the researchers. Whenever possible, the 28‐day subsample consisted of 28 consecutive days in the middle of the exercise treatment period. In instances of missing data due to the reasons listed previously, the gaps were filled with the recorded, immediately adjacent running data.

Mean running distance was calculated as the average kilometers run per day across the 28‐day sample for each mouse. Active time was calculated as the number of 1‐min bins during which the mouse was participating in wheel running. Running velocity was reported as the mean wheel rotations per minute for every 1‐min bin during which the mice was active. Running bursts were defined as consecutive 1‐min bins during which the mouse was active while rests were defined as consecutive 1‐min bins during which the mouse was inactive. Latency to run is defined as the mean amount of time after dark onset until a mouse engaged in wheel running. Latency to stop is defined as the mean amount of time after light onset until a mouse stopped engaging in wheel running.

### Glucose tolerance

An intraperitoneal glucose tolerance test (IPGTT) was performed on mice at approximately 6 months of age following completion of 5 of the 6 months of voluntary exercise (for mice with wheel access). Mice were fasted for 12 h through their dark phase (0800–2000 h) prior to administration of the IPGTT. Mice were injected with a volume of 25% glucose solution equivalent to 1 g of glucose per kg of bodyweight (University of Virginia Vivarium Protocols, Susanna R. Keller). A small incision was made on the tail and blood samples were collected with an Ascensia CONTOUR^TM^ Blood Glucose Monitoring System (Bayer Healthcare, Whippany, NJ, https://www.contournext.com/products/contour-next/) paired with CONTOUR^TM^ Blood Glucose Test Strips (Bayer Healthcare) to determine blood glucose levels at baseline (prior to injection) and at set timepoints 10, 20, 30, 60, 90, and 120 min following the injection. Mice in voluntary exercise treatment groups retained access to their running wheel for the duration of the IPGTT.

### Statistical analysis

Final body weight, weight of adipose tissue, integrated area‐under‐the‐curve (iAUC) analysis of glucose tolerance, and all the calculated running behaviors (distance, velocity, bursts, etc.) were compared using an analysis of variance (ANOVA). All statistical tests used *α* = 0.05 as the minimum confidence interval for significance and all reported values are mean ± standard deviation (s.d.). All post hoc analyses utilized the Tukey’s multiple comparison test and significantly different mean‐wise comparisons were indicated by different letters in the figures. Data organization and analysis were performed with Microsoft Excel and The R Project statistical computation system (R Core Team 2018). Double‐plotted actograms were generated using Fiji and the associated plugin ActogramJ (http://actogramj.neurofly.de/) (Schmid et al. 2011; Schindelin et al. 2012). Graphs were designed and generated using Origin Student 2018b software (version b9.5.5.409 OriginLab Corporation, Northhampton, MA, https://www.originlab.com/index.aspx?go=PRODUCTS/OriginStudentVersion). Statistical tests were performed with Graphpad Prism (version 7.04, Graphpad Software, La Jolla, CA, https://www.graphpad.com/scientific-software/prism/).

## Results

All data, underlying the results and reported therein, are available upon request from the authors.

### Wheel running offers partial protection from the effects of MHF diet

**Figure 1:**
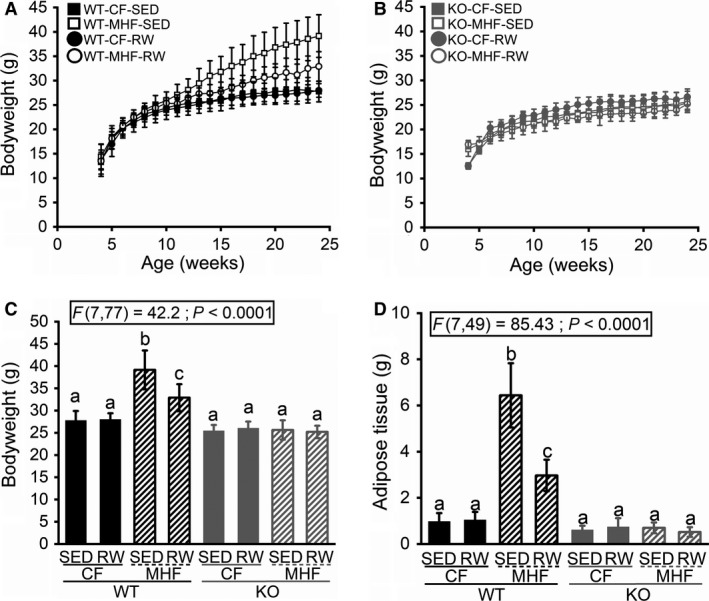
Bodyweight and deposition of adipose tissue is not altered by maintenance on a moderately high‐fat diet or access to voluntary running wheels in K_v_1.3‐null mice. (A and B) Line graphs of the average bodyweight over time for wildtype (WT) and K_v_1.3‐null mice (KO) with varying diets and access to exercise starting at 4 weeks of age (weaning) and continuing through 24 weeks. Control diet (CF; 13.5% fat), moderately high‐fat (MHF; 32% fat), sedentary SED), voluntary running wheel (RW) (C) Bar graph of the bodyweight (at 24 weeks of age) for each treatment group. (D) Bar graph of the adipose tissue weight collected upon manual excision following fix perfusion. Data are reported as mean (Standard Deviation, SD); One‐Way analysis of variance (ANOVA) where different letters represent significantly‐different means as determined by Tukey's multiple comparison *Post hoc* test. Mice (males only) per treatment group as follows: (A) 18, 12, 15, and 10. (B) 7, 7, 6, and 10. (C) 18, 15, 12, 10, 7, 6, 7, and 10. (D) 9, 9, 7, 5, 6, 5, 6, and 10.

**Figure 2:**
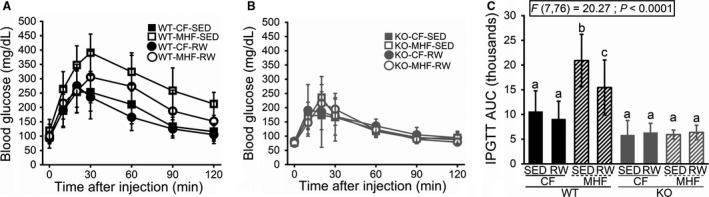
Ability to clear a glucose challenge is not affected by diet or access to voluntary exercise wheels in K_v_1.3‐null mice. (A and B) Line graphs of blood glucose levels after fasting (0 min) versus a glucose challenge over time up to 2 h. (C) Bar graph of the integrated area under the curve (AUC) for the graphs shown in A + B. Data are reported and applied statistical metric and notations are as in Figure 1. Mice (males only) per treatment group as follows: (A) 17, 12, 15, and 10. (B) 7, 7, 6, and 10. (C) 17, 15, 12, 10, 7, 6, 7, and 10.

WT mice fed the CF diet weighed the same regardless of whether they had access to a running wheel (WT‐CF‐SED 24‐week bodyweight = 27.8 g (SD 2.1); WT‐CF‐RW = 28.0 (SD 1.4)). Mice fed the MHF diet without access to a running wheel gained significantly more bodyweight than all other treatment groups (WT‐MHF‐SED = 39.2 g (SD 4.4); F(7,77) = 42.2; *P* < 0.0001). Mice fed the MHF diet with access to a running wheel weighed significantly less than the MHF‐fed mice without a running wheel (WT‐MHF‐RW = 32.9 g (SD 3.0) but they still weighed significantly more than the CF‐fed treatment groups (Fig. 1A and C). The bodyweight of KO mice was not affected by wheel access or diet modification (KO‐CF‐SED = 25.4 g (SD 1.3), KO‐CF‐RW = 26.1 (SD 1.5), KO‐MHF‐SED = 25.6 (SD 2.2), KO‐MHF‐RW = 25.2 (SD 1.4)) (Fig. 1B and C). The same pattern was present for the adiposity of the mice. WT mice fed the CF diet displayed similar amounts of adipose tissue regardless of wheel access (WT‐CF‐SED = 0.97 g (SD 0.37); WT‐CF‐RW = 1.04 g (SD 0.35)). And the MHF‐fed mice without a wheel displayed the most adiposity while the MHF‐fed mice with wheel access were intermediate, with significantly more adipose tissue than the CF‐fed groups and significantly less adipose tissue than the MHF sedentary mice (WT‐MHF‐SED = 6.44 g (SD 1.39); WT‐MHF‐RW = 2.97 (SD 0.68); F(7,49) = 85.43; *P* < 0.0001) (Fig. 1D). KO mice displayed similar amounts of adipose tissue regardless of wheel access or diet modification (KO‐CF‐SED = 0.61 g (SD 0.18); KO‐CF‐RW = 0.74 (SD 0.38), KO‐MHF‐SED = 0.70 (SD 0.23), KO‐MHF‐RW = 0.52 (SD 0.21)) (Fig. 1D). KO mice displayed the slim body type and reduced adiposity reported in prior experiments (Fadool et al. 2004), but this effect did not reach statistical significance in our current cohort. This pattern is replicated again in the results of the IPGTT (Fig. 2). Again, WT mice maintained on the CF diet with or without wheel access showed no differences in their ability to clear glucose, as assessed by calculating the area under the curve of their respective glucose tolerance curves (WT‐CF‐SED = 10,589 (SD 4196); WT‐CF‐RW = 9073 (SD 3624)). Significant glucose intolerance was present in the MHF‐fed mice without wheel access while the MHF‐fed mice with a wheel again had an intermediate value between the CF treatment groups and the MHF sedentary group (WT‐MHF‐SED = 20,922 (SD 5313); WT‐MHF‐RW = 15,462 (SD 5572); F(7,76) = 20.27; *P* < 0.0001). All KO treatment groups displayed similar glucose clearance rates regardless of wheel access or diet, exhibiting the increased glucose sensitivity compared to WT previously reported (Thiebaud et al. 2014), but again not reaching statistical significance in our current cohort (KO‐CF‐SED = 5839 (SD 2945); KO‐CF‐RW = 6348 (1865), KO‐MHF‐SED = 5932 (SD 923), KO‐MHF‐RW = 6367 (SD 1474)).

### Wheel running is consistent over time independent of genotype or diet

**Figure 3:**
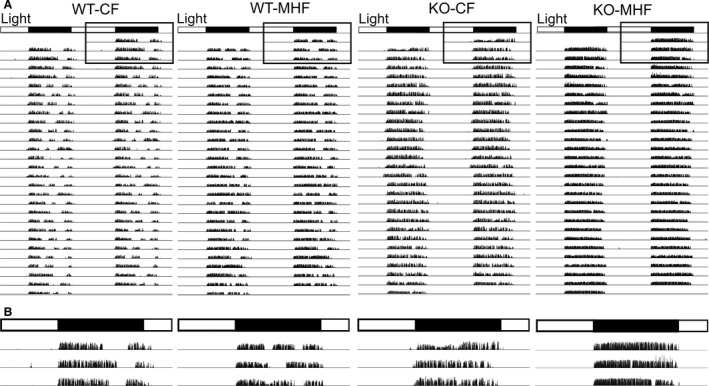
Running behavior can be visualized and qualitatively assessed through use of a double‐plotted actogram. (A) Representative of a 28‐day sample of running wheel activity for one mouse in each treatment group. Each spike corresponds to a 1‐min time block and the amplitude of the spike is determined by how many wheel rotations occurred in that 1‐min bin. (B) Inset box from (A) Magnified view of 3 days of running wheel activity for each treatment group. Open box = light cycle, closed box = dark cycle.

**Figure 4:**
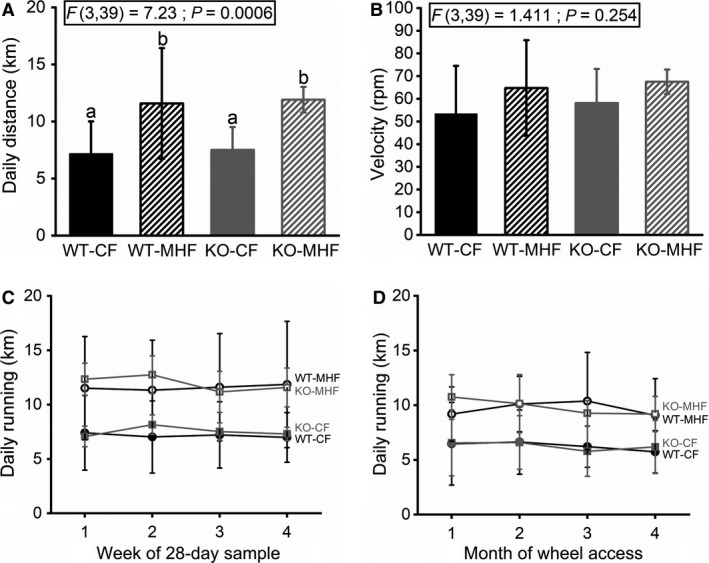
Running velocity and distance are consistent over time for CF and MHF chronically‐maintained WT and KO mice. Mice maintained on MHF diet run further, but not greater velocity, irrespective of genotype. Bar graph of (A) Running distance and (B) Velocity computed over a 28‐day interval. (C and D) Line graph of the mean daily running distance over time in (C) weeks and (D) months, respectively. Data are reported and applied statistical metric and notations are as in Figure 1. Mice (males only) per treatment group as follows: (A–D) 15, 10, 10, and 8.

**Figure 5:**
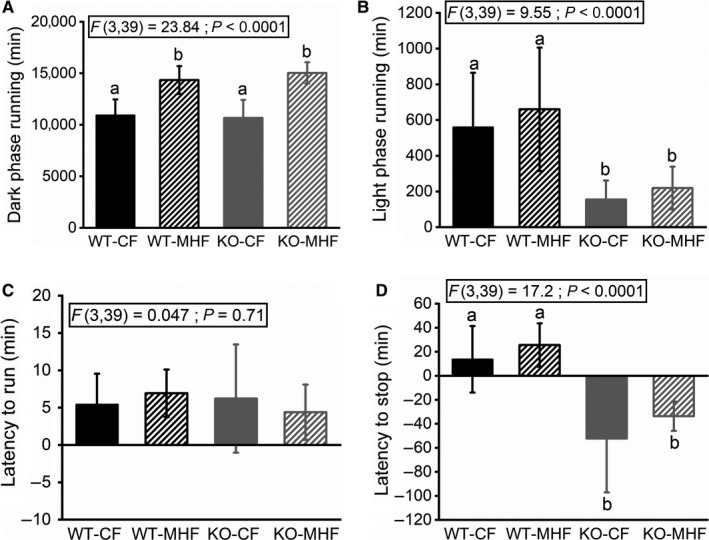
Light and dark phase running patterns in WT and KO mice maintained on CF versus MHF diets. (A and B) Bar graphs of active wheel running in the (A) dark and (B) light phase, respectively, across a 28‐day sample. (C) Bar graph of the latency to first run following dark onset (D) Bar graph of the latency to stop running following light onset. Data are reported and applied statistical metric and notations are as in Figure 1. Mice (males only) per treatment group (A–D) 15, 10, 10, and 8.

**Figure 6:**
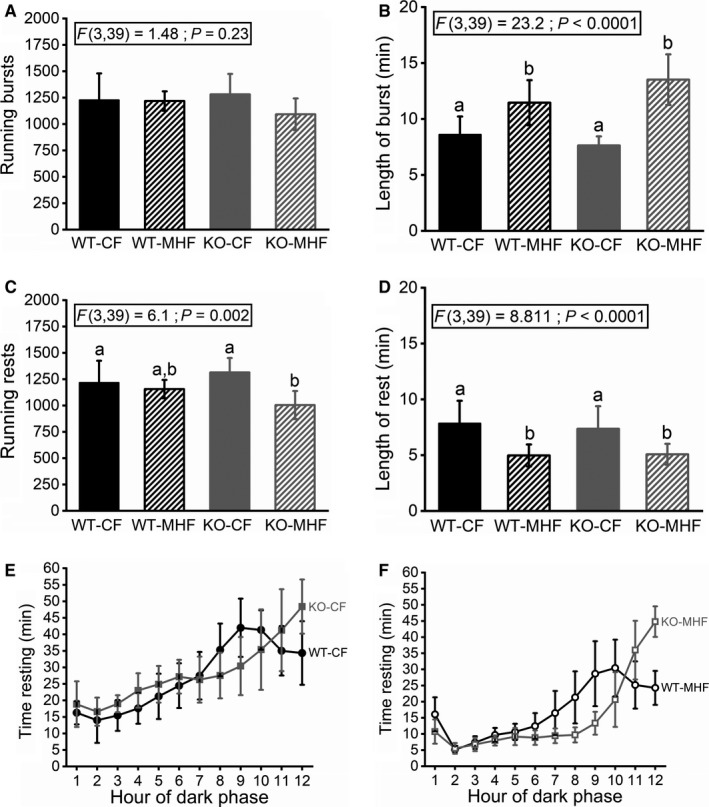
Running bursts and rest intervals observed in WT and KO mice maintained on CF versus MHF diets. Bar graph comparing (A) Number of running bursts, (B) Burst length, (C) Number of rest intervals, and (D) Rest length computed over a 28‐day interval. (E and F) Line graph plotting resting time versus the 12‐h dark phase in a cohort of (E) CF‐ and (F) MHF‐challenged mice, respectively at the 4‐month time point. Data are reported and applied statistical metric and notations are as in Figure 1. Mice (males only) per treatment group (A–D) 15, 10, 10, and 8. (E) 15 and 10. (F) 12 and 8.

Following the 3‐day acclimation period and barring any technical errors, mice in all treatment groups that were provided a wheel ran very consistently over the full 120‐160 day running period. Qualitative assessment of double‐plotted actograms showed that mice participate in wheel running in a manner that is fairly entrained to their light/dark cycle without gross deviation (Fig. 3). Quantification of the latency to stop running upon the termination of the dark cycle was found to uncover differences across genotypes in the time‐locked behavior to the actogram, not related to diet (Fig. 5, see results below). Tracking the mean daily running distance over time showed that on a weekly and monthly basis, the mice ran consistently (Fig. 4C–D). The data in Figures 4, 5, 6 contain refined running analyses, and therefore we removed the prefix “RW” (running wheel) from the graphs and text for clarity, because all mice in these figures are wheel runners.

### K_v_1.3 ablation and fatty diet affect wheel running activity

Both WT and KO mice maintained on the MHF diet ran farther than the two CF‐fed groups (WT‐CF = 7.16 km (SD 2.85); KO‐CF = 7.51 (SD 2.0); WT‐MHF = 11.58 (SD 4.85); KO‐MHF = 11.91 (SD 1.12); F(3,39) = 7.23, *P* = 0.0006) (Fig. 4A). The WT‐MHF and KO‐MHF mice ran slightly faster than the WT‐CF and WT‐MHF but this did not reach statistical significance (Fig. 4B).

Across the 28‐day sample of wheel running data, there are a total of 40,320 min, split evenly between the light and dark phases. We tracked how many minutes the mice were actively participating in wheel running in the light and dark phases. The MHF‐fed mice spend more time running than the CF‐fed mice across both genotypes (WT‐CF = 10,927 min (SD 1541), KO‐CF = 10,674 (SD 1754), WT‐MHF = 14,347 (SD 1358), KO‐MHF = 15,039 (SD 1034); F(3,39) = 23.84, *P* < 0.0001) (Fig. 5A). In the light phase, the WT mice spent more time running than the KO mice across both diet treatments (WT‐CF = 560 (SD 305), WT‐MHF = 660 (SD 346), KO‐CF = 155 (SD 107), KO‐MHF = 219 (SD 120); F(3,39) = 9.55, *P* < 0.0001) (Fig. 5B).

We measured both the latency to start wheel running after the onset of the dark phase (Fig. 5C) and the latency to stop wheel running after the onset of the light phase (Fig. 5D). All treatment groups began running soon after the onset of the dark phase (Fig. 5C) and showed no difference in this behavior (F(3,39) = 0.047, *P* = 0.71). WT mice continued running after the onset of the light phase whereas KO mice stopped running even before light onset (WT‐CF = 13.7 active minutes after light onset (SD 27.7), WT‐MHF = 25.6 (SD 18.1), KO‐CF = −52.3 (SD 44.8), KO‐MHF = −33.6 (SD 12.2); F(3,39) = 17.2, *P* < 0.0001) (Fig. 5D). The negative numbers here reflect that the KO mice stopped running approximately 30 to 60 min prior to the onset of the light cycle, irrespective of diet.

Running bursts were calculated as the number of consecutive 1‐min bins that a mouse was actively engaged in wheel running during the dark phase. Rests were calculated similarly as the number of consecutive 1‐minute bins during which the mouse was not actively running during the dark phase. Thus, the length of a burst or rest could range from 1‐min long to hours. All four treatment groups engaged in the same number of bursts (WT‐CF = 1227 bursts (SD 252); WT‐MHF = 1218 (SD 93); KO‐CF = 1282 (SD 194), KO‐MHF = 1093 (SD 150); F(3,39) = 1.48, *P* = 0.23), but the length of the running bursts were significantly longer for MHF‐fed mice compared to CF‐fed mice for both genotypes (WT‐CF = 8.6 min per burst (SD 1.6), KO‐CF = 7.6 (SD 0.8), WT‐MHF = 11.5 (SD 2.0), KO‐MHF = 13.5 (SD 2.3); F(3,39) = 23.2, *P* < 0.0001) (Fig. 6A + B). KO‐MHF mice engaged in the fewest rests, significantly less than the CF‐fed groups but not statistically significant compared to the WT‐MHF group (WT‐CF = 1,217 (SD 208), WT‐MHF = 1156 (SD 87), KO‐CF = 1315 (SD 136); KO‐MHF = 1005 (SD 134); F(3,39) = 6.1, *P* = 0.002) (Fig. 6C). Both MHF‐fed groups engaged in shorter rests compared to the two CF‐fed groups (WT‐CF = 7.9 min per rest (SD 2.0); KO‐CF = 7.4 (SD 2.0); WT‐MHF = 5.0 (SD 1.0), KO‐MHF = 5.1 (SD 0.9); F(3,39) = 8.8, *P* < 0.0001)(Fig. 6D). We examined the distribution of resting behavior across the dark phase by binning the activity into hours. Independent of diet, both WT groups had an average peak resting period of approximately 40 min per hour at 10 h into the dark phase, which shortened toward the end of the dark phase, with concomitant increased activity. The KO mice, however, never exhibited such a plateau and steadily increased their rests periods incrementally throughout the dark phase up through the transition to the light phase where they rarely ran (Fig. 6E + F and Fig. 5B + D).

## Discussion

These studies sought to investigate the effects of diet modification and genetic deletion of the voltage‐gated potassium channel K_v_1.3 on voluntary exercise. K_v_1.3‐null (KO) mice ran the same distance and speed as WT mice when they were maintained on the same diet. However, the patterns of running activity and resting were different in the two genotypes. Both genotypes were highly active on their wheels in the early portion of the dark phase, spending little time resting. Both genotypes also steadily increased the amount of resting as the dark phase progressed. WT mice rested the most during hours 9 and 10, increasing their activity again in the last 2 h of the dark phase while the KO mice did not exhibit this plateau, resting most heavily during hours 11 and 12 of the dark phase. In fact, the WT mice continued running even after the onset of the light phase while the KO mice seemingly predicted the transition to the light phase, ceasing their running behavior even before light onset. Previous studies have demonstrated increased energy expenditure and locomotor activity KO mice, but this is a novel characterization of their voluntary exercise phenotype (Tucker et al. 2008). The data presented here do not offer direct evidence of the potential mechanism for the different wheel running patterns of the KO mice, but there are a number of possibilities. The KO mice exhibit a “super smeller” phenotype so it is possible that increased olfactory exploration may interact with the timing of their wheel running activity (Fadool et al. 2004). The KO mice are also resistant to mitochondrial alteration following challenge with MHF diet as well as increased respiratory exchange ratio, hinting that energy utilization is altered in the KO mice, which may in turn influence their wheel running patterns (Upadhyay et al. 2013; Kovach et al. 2016).

We also observed alterations of wheel running behavior following diet modification. Both WT and KO mice ran farther distances daily when maintained on the MHF diet compared to the CF diet. We determined that, in both genotypes, the increased daily running distance is primarily driven by MHF‐fed mice participating in longer bursts of running and shorter rests compared to CF‐fed mice. The MHF‐fed mice did run slightly faster than their CF‐fed counterparts, but this was not statistically significant. This is interesting because the body of literature is fairly split on this topic. Vellers et al. (2017) observed reduced time spent running, running distance, and running velocity in mice fed a high‐fat, high‐sugar diet in both short‐ and long‐term feeding. It is worth noting that in this experiment short‐term is defined as a 3‐day period of access to the high‐fat, high‐sugar diet and the long‐term exposure was 14 days. Other studies have demonstrated similar reductions in mouse wheel running following maintenance on various high‐fat diets while both mouse and human studies have demonstrated that increased caloric intake reduces spontaneous physical activity (Bjursell et al. 2008; Levine et al. 2008; Schmidt et al. 2012). However, some investigators have observed an increase in wheel running following exposure to high‐fat diet. For example, Meek et al. (2010), noted an increase in wheel running in a line of mice selective bred for high levels of voluntary exercise following a switch to a “Western diet.”. Furthermore, some studies have noted genotype‐specific alterations of voluntary exercise, noting changes in some lines but not others following diet modification (Funkat et al. 2004). The contrasting effects observed between previous studies and the results we report here may be due to the amount of time the mice are maintained on the modified diet, the composition of the diet, or slight differences between the lines of mice being studied.

Participation in voluntary exercise partially ameliorated the negative metabolic effects of consumption of the MHF diet. WT mice with running wheel access maintained on the MHF diet showed lower bodyweight, lower adiposity, and improved glucose tolerance compared to WT mice on the same diet without a wheel. Because adiposity and glucose tolerance were only determined at a single time point, we cannot resolve whether these metrics steadily improved with time. It is worth discussing, as a limitation to the interpretation of our data, that the voluntary exercise treatment groups retained access to their running wheels during the fasting period leading up to and for the duration of the IPGTT. This means we could not control the acute effects of an exercise bout prior to or during the IPGTT. We did this to avoid potential stressors associated with removal of the running wheel. Previous studies have shown increases in anxiety‐ and depressive‐like behaviors in mice following cessation of voluntary exercise (Malisch et al. 2009; Nishijima et al. 2013). Our results, however, replicated what has been demonstrated by previous studies investigating combinatorial effects of diet and exercise modification. Mice with access to a running wheel consistently display better metabolic health when maintained on MHF diet, but the benefits of the wheel are not as effective as removal of the MHF diet (or of never being exposed to the MHF diet to begin with). Thus, the high‐fat fed mice given wheel access typically display an intermediate level of metabolic health that is between MHF non‐runners and CF fed mice. Hicks et al. (2016) found lower weight gain and lower levels of glucose intolerance in mice fed a 60% kcal from fat diet after 11 weeks in the group that had running wheel access compared to the group that only had access to a locked wheel. Yoshimura et al. (2018) observed that maintenance on a 57% kcal from fat diet induced accumulation of liver fat that was reduced following 1 week of wheel running and other studies have displayed similar results of at least partial rescues of varying negative metabolic phenotypes. The KO mice are already resistant to weight gain, adipose deposition, and glucose intolerance and we did not observe any differences in these metabolic parameters regardless of diet or wheel access in the KO mice. It is interesting that we did not see improved glucose tolerance in the KO mice that were provided a wheel compared to the KO mice that were not. It is known that protein kinase B activity is increased following exercise leading to increased GLUT4 translocation in skeletal muscle (Sakamoto and Holman 2008). There is some evidence that K_v_1.3 influences GLUT4 also via the protein kinase B pathway (Jaimes‐Hoy et al. 2017). It is possible that the wheel running and the genetic deletion of K_v_1.3 are influencing glucose uptake via the same signaling pathway but in a non‐additive manner.

Our study has revealed that KO mice exhibit a voluntary exercise pattern that is different than WT mice in regard to their patterns of rest, especially later in the dark phase. We have also demonstrated that both WT and KO mice, when maintained on an MHF diet for 6 months, increase their levels of voluntary exercise compared to mice maintained on the CF diet. Understanding how these two variables affect exercise behaviors is important because of the growing potential of K_v_1.3 as a therapeutic target. We believe it is especially interesting because of the role that K_v_1.3 plays in the inflammatory response and the recent hypothesis that obesity is a state of chronic low‐grade inflammation (Reilly et al. 2017). This means that K_v_1.3‐targeted therapies, exercise regimens, and diet modification could be used in conjunction to treat metabolic disorders eventually in humans, so it is important to understand how these treatments interact. One feature of other treatments targeted toward metabolic syndrome (metformin, rosiglitazone, etc.) is that they negatively impact exercise performance or lead to complications when combined with increased physical activity. Our data suggest that treatments targeted toward K_v_1.3 might not suffer from this same drawback. Though the different running patterns of the KO mice mentioned above are almost certainly not the driver of DIO resistance or improved glucose tolerance, it is still promising for researchers interested in exploring K_v_1.3‐targeted treatments as there was no decline in the exercise capacity measured by running velocity or running distance.

Finally, our results suggest that it might be important for researchers using transgenic rodent models to monitor the running behavior of their animals in finer detail than is typically reported. The amount of exercise that mice perform each day is highly variable depending on the transgenic line being used (Castilla‐Ortega et al. 2013). Furthermore, some studies do not even report the amount of exercise and seem to assume that all mice are running normally simply because a wheel is present in their cage. If investigators only quantify daily activity (or do not quantify activity at all), they could be missing differences in exercise patterns that are potentially physiologically relevant.

## Conflict of Interest

The authors have no conflicts of interest, scientific or financial.
